# Autonomic Influence on Heart Rate for Deep Breathing and Valsalva Maneuver in Healthy Subjects

**Published:** 2018-06-30

**Authors:** Reena Kumari Jha, Amrita Acharya, Ojashwi Nepal

**Affiliations:** 1 Department of Physiology, Kathmandu University School of Medical Sciences, Dhulikhel, Kavre, Nepal; 2 Human Biology, Kathmandu University, Dhulikhel, Kavre, Nepal

**Keywords:** *autonomic nervous system*, *deep breathing test*, *heart rate*, *valsalva maneuver*

## Abstract

**Introduction:**

The Autonomic nervous system is responsible for regulation and integration of visceral functions. Disturbance of autonomic nervous system play crucial role in pathogenesis and clinical course of many diseases. In the present study deep breathing test and valsalva maneuver have been described to monitor parasympathetic function genderwise.

**Methods:**

A cross-sectional study was conducted among 100 subjects, aged 18-25 years, from May to November 2017, in exercise physiology laboratory, Kathmandu University School of Medical Sciences, Chaukot, Kavre. Electrocardiograph recorded by AD instrument was used to calculate the resting heart rate and the heart rate response to deep breathing test and valsalva maneuver.

**Results:**

Heart rate response to deep breathing test (31.69+14.79 Vs. 36.08+18.65, P=0.195) and valsalva ratio (1.59+0.39 Vs. 1.69+0.54, P=0.314) tend to be higher in female than male subjects but not significant. The resting heart rate of females was significantly higher than that of males (84.37 + 11.08 Vs. 78.43 + 12.06, P<0.05). Heart rate was significantly increased during and decreased after valsalva maneuver in both male and female subjects.

**Conclusions:**

This study concludes that both deep breathing test and valsalva maneuver activates parasympathetic system inhealthy subjects. And also dominant parasympathetic activity was found in female comparison to male subjects.

## INTRODUCTION

The parasympathetic and sympathetic division of autonomic nervous system (ANS) work in a coordinated manner to integrate and regulate vital functions at subconscious level.^1,2^ This helps in survival and enabling the body to cope with exercise, stress and other adaptive responses.^3^

Assessment of autonomic nervous functions provides important information about autonomic status in health and disease. Autonomic failure is reported inmany clinical conditions such as myocardial infarction, diabetes mellitus, alcoholism, kidney disease and clinical symptoms may appear late in course of disease.^4^

The assessment of ANS can be made using several non-invasive methods such as deep breathing, valsalva maneuver, handgrip, cold pressor test, lying to standing test or head up tilt test.^1,2^ The disruption of parasympathetic nervous system is usually detected earlier than that of the sympathetic system.^4^ Thus the present study aims to evaluate the autonomic nervous function in healthy undergraduates according to gender.

## METHODS

A cross sectional study was carried among undergraduates of Kathmandu University School of Medical sciences, Chaukot, Kavre. Recruitment was started from May 2017 to November 2017 after taking approval from institutional review committee of Kathmandu University School of Medical Sciences/ Dhulikhel Hospital (IRC-KUSMS). Sample was collected randomly from the population. Subjects suffering with cardiorespiratory disease and autonomic dysfunction were excluded from the study. Sample size can be calculated by following formula

$$\text{n=}{\left(\text{Zα/2 + Zβ}\right)}^{2}\left({\text{σ1}}^{2}{\text{+σ2}}^{2}\right){\text{/σ}}^{2}$$

where n is the sample size, z is the z score, α is level of significance, β is type II error probability, σ1^2^standard deviation of previous deep breathing test, σ2^2^standard deviation of previous valsalva ratio and δ^2^ is error in terms of absolute value.

$$\begin{array}{l} \text{Male; n = }{\left(\text{Zα/2 + Zβ}\right)}^{2}\left({\text{σ1}}^{2}{\text{ + σ2}}^{2}\right){\text{ / σ}}^{2}\\ \text{=}{\left(1\text{.96 + 1}.28\right)}^{2}\left\{{\left(8.5\right)}^{2}\text{ + }{\left(0.2\right)}^{2}\right\}\text{/ }{\left(3.8\right)}^{2}\\ \text{=}52\\ \text{Female; n = }{\left(\text{Zα/2 + Zβ}\right)}^{2}\left({\text{σ1}}^{2}{\text{ + σ2}}^{2}\right){\text{ / σ}}^{2}\\ \text{=}{\left(1\text{.96 + 1}.28\right)}^{2}\left\{{\left(10.5\right)}^{2}\text{+ }{\left(0.2\right)}^{2}\right\}\text{/ }{\left(4.8\right)}^{2}\\ \text{=50} \end{array}$$

First the subject was informed about the procedure and consent was taken. Then the subject was asked to take rest for 5 minutes. At the mean time electrodes were placed on proper position of the body. The baseline data was recorded for one minute. The parameters that were used to test the autonomic functions consist of two tests employing heart rate response reflecting parasympathetic functions are: Heart rate response to deep breathing (HR DBT) and Heart rate response to valsalvamaneuver (VR).

Heart rate response to deep breathing: The subject was asked to lie down on the bed comfortably and relax. The subject was asked to breathe slowly, smoothly and deeply at the rate of 6 breathes per minute, guided by hand signal for a period of one minute. One respiratory cycle lasts for 10 seconds, 5 seconds for each inhalation and exhalation. A continuous ECG was recorded for one minute. The mean of the minimum R - R interval during inspiratory cycles was calculated and the heart rate was determined. The mean of the maximum R - R interval during expiratory cycles was calculated and the heart rate was determined. The changes in heart rate were calculated as the difference between maximum and minimum heart rate. The result was then expressed as beats per minute.

Heart rate response to valsalva maneuver: The subject was asked to seat comfortably then blow into a mouthpiece connected to a mercury sphygmomanometer to the pressure level of 40 mm Hg for 15 seconds. ECG changes were recorded throughout the maneuver and 30 seconds after the procedure. The result was expressed at the valsalva ratio, which is the ratio of the longest R - R interval after the valsalva maneuver to the shortest R - R interval during the valsalva maneuver.

Data were entered into Microsoft EXCEL and analyzed using SPSS version 22 software. Student ‘t’ test was used to analyze basal heart rate with heart rate during valsalva maneuver and basal heart rate with heart rate after valsalva maneuver in male and female respectively. P value less than 0.05 is considered significant.

## RESULTS

A total of 100 undergraduates were enrolled and analysed. 50% were male and 50% were female. The age range of participants was 18 - 25 years with mean age of male (19.98±1.42) and female (19.74±1.20). We did not observe any significant difference between male and female in term of heart rate response to deep breathing and valsalva ratio.

The baseline heart rate was significantly higher in female compared to male (84.37±11.08 vs. 78.43±12.06; P=0.012). During valsalva maneuver, heart rate was increased and after valsalva maneuver, heart rate was decreased from baseline level in both male and female subjects.

The heart rate response to deep breathing test was calculated as the difference between maximum heart rate during inhalation and minimum heart rate during exhalation. A difference of 15 beats/min or more is considered parasympathetic activation. Valsalva ratio is the ratio of the longest R - R interval after the valsalva maneuver to the shortest R - R interval during the valsalva maneuver. A value of 1.21 or more is defined as normal response. ^5^ In both male and female subject's heart rate response to deep breathing test and valsalva ratio showed parasympathetic activation as shown (Table 1). While comparing between male and female, the difference was insignificant.

**Table 1.** Autonomie influence of DBT and VM on heart rate gender wise.

Variable	Male Mean ±SD	Female Mean ±SD	P
HR DBT	31.69±14.79	36.08±18.65	0.195
Valsalva ratio	1.59±0.39	1.69±0.54	0.314

Heart rate was significantly increased during valsalva maneuver and decreased after valsalva maneuver in both male and female subjects (Table 2 and 3 respectively).

**Table 2.** Comparison of basal HR with HR during and after VM in Male.

Variable	Mean±SD	P
HR Initial & During VM	-11.36±12.89	0.000
HR Initial & After VM	6.66±6.53	0.000

**Table 3.** Comparison of basal HR with heart rate during and after VM in Female.

Variable	Mean±SD	P
HR Initial & During VM	-12.18±16.57	0.000
HR Initial & After VM	7.44±6.51	0.000

HR was significantly higher in female than male at basal level, during as well as after VM. There was significant difference between genders in terms of HR at rest, during and after VM depicted (Table 4).

**Table 4.** Comparison of HR response before, during and after VM according to gender.

Variable	Male Mean±SD	Female Mean±SD	P
Initial HR	78.43±12.06	84.37±11.08	0.012
During VM HR	89.80±15.91	96.55±17.92	0.049
After VM HR	71.76±7.85	76.93±9.55	0.004

## DISCUSSION

Balance between sympathetic nervous system and parasympathetic nervous system, two branches of autonomic nervous system, regulates heart rate. The specific activity of each branch of autonomic nervous system can be estimated by different non-invasive methods such as head-up tilt, cold pressure test, deep breathing and Valsalva maneuver.^2^ The Valsalva maneuver is a forced expiratory effort against a closed airway and shows two responses: intrastrain tachycardia and post strain bradycardia.^6^ VM has a wide range of applications in several medical disciplines including diagnosing heart problems or autonomic nervous system deficiencies.^7^ Deep breathing refers to the prolonged inhalation and exhalation. It is one of the simple and powerful relaxation techniques that is designed to improve the efficiency of ventilation, decreases the work of breathing and improves gas exchange.^8^

Heart rate response to deep breathing test and Valsalva ratio was insignificantly higher in female than male. This finding was consistent with study conducted by Piha who concludes that heart rate response to valsalva ratio was greater in females over 50 years than in males of the same age and the heart rate response to deep breathing was higher in females under 50 years than in males under 50 years.^9^ This may be due to the developmental difference or the effect of the prevailing levels of male and female sex hormones.^10^ Estrogen have facilitatory effect on cardiovagal function. Oestrogen increases the density and activity of muscarinic receptors thereby increasing the vagal tone and suppressing sympathetic activity.^11^ Ovariectomized female animals showed enhanced sympathetic activation compared with intact female animal and differences were minimized by oestrogen treatment. ^12^

The present study showed that there is significant difference in resting heart rate between genders. The females have significantly higher resting heart rate than males. This result is consistent with a study where resting heart rate is significantly higher in females aged under 50 years than in males of the same age.^9^ Burke et al demonstrated that young adult women have faster resting heart rate than healthy adult male. The potential mechanisms include direct or indirect hormonal effects on the electrical properties of the sinus node, chronic developmental differences in sinus node function, differences in stroke volume; differences in autonomic influences on the sinus node.^13^ On other hand Sheila observed no difference in basal mean heart rate between male and female at any age.^14^

In practice, the valsalva maneuver has four phases: phase I - starting of forced expiration; phase II -continuous straining with sustained blow; phase IIIrelease of respiratory strain; phase IV - immediately after release of respiratory strain.^6^

The present study revealed that during VM (phase II, III - Intrastrain) heart rate was increased and after the valsalva maneuver (phase IV- post strain) heart rate decreased in both male and female subjects. This result is parallel with several other studies.^15,16,17^ Straining for 15 seconds increases the intrathoracic pressure (intrathoracic pressure become higher than pressure in great vein) and intraabdominal pressure that results decrease in venous return to the heart with an increase in venous pressure, progressive arterial pressure reduction, and consequently, progressive compensatory baroreflex ex-mediated heart rate increases.^15^ Following cessation of the straining phase, the functional changes that occurred during straining were abruptly reversed, resulting in an overshoot of arterial pressure accompanied by a rapid and progressive baroreflex mediated decrease in heart rate. During this period the sympathetic activity is low.^18^

## CONCLUSIONS

The present study showed that both deep breathing test and valsalva maneuver activates parasympathetic nervous system in healthy subjects. Comparing between genders, the parasympathetic activity was found to be higher in females than in males. The intricate age-gender interaction in the autonomic control of the heart and its relationship to cardiovascular diseases warrant further exploration.
